# Decellularized Wharton Jelly Implants Do Not Trigger Collagen and Cartilaginous Tissue Production in Tracheal Injury in Rabbits

**DOI:** 10.3390/life12070942

**Published:** 2022-06-23

**Authors:** Katia Martins Foltz, Aloysio Enck Neto, Júlio César Francisco, Rossana Baggio Simeoni, Anna Flávia Ribeiro dos Santos Miggiolaro, Thatyanne Gradowski do Nascimento, Bassam Felipe Mogharbel, Katherine Athayde Teixeira de Carvalho, José Rocha Faria-Neto, Lúcia de Noronha, Luiz César Guarita-Souza

**Affiliations:** 1 Graduate Program in Health Sciences, School of Medicine, Pontifícia Universidade Católica do Paraná (PUCPR), Curitiba 80215-901, Paraná, Brazil; aloysioenck@gmail.com (A.E.N.); thaty.gfcn@gmail.com (T.G.d.N.); jose.faria@pucpr.br (J.R.F.-N.); lnno.noronha@gmail.com (L.d.N.); guaritasouzalc@hotmail.com (L.C.G.-S.); 2 Experimental Laboratory of Institute of Biological and Health Sciences, Pontifícia Universidade Católica do Paraná (PUCPR), Curitiba 80215-901, Paraná, Brazil; julio.apfr@gmail.com (J.C.F.); rossanabaggio@gmail.com (R.B.S.); annafla007@hotmail.com (A.F.R.d.S.M.); 3 Advanced Therapy and Cellular Biotechnology in Regenerative Medicine Research Group, Pelé Pequeno Príncipe Research Institute & Pequeno Príncipe Faculties (FPP), Curitiba 80250-060, Paraná, Brazil; bassamfm@gmail.com (B.F.M.); katherinecarv@gmail.com (K.A.T.d.C.); 4 Laboratory of Experimental Pathology, Graduate Program of Health Sciences, School of Medicine, Pontifícia Universidade Católica do Paraná (PUCPR), Curitiba 80215-901, Paraná, Brazil

**Keywords:** scaffold, biomaterial, tissue engineering

## Abstract

Background: Tracheal lesions are pathologies derived from the most diverse insults that can result in a fatal outcome. Despite the number of techniques designed for the treatment, a limiting factor is the extent of the extraction. Therefore, strategies with biomaterials can restructure tissues and maintain the organ’s functionality, like decellularized Wharton’s jelly (WJ) as a scaffold. The aim is to analyze the capacity of tracheal tissue regeneration after the implantation of decellularized WJ in rabbits submitted to a tracheal defect. Methods: An in vivo experimental study was undertaken using twenty rabbits separated into two groups (*n* = 10). Group 1 submitted to a tracheal defect, group 2 tracheal defect, and implantation of decellularized WJ. The analyses were performed 30 days after surgery through immunohistochemistry. Results: Inner tracheal area diameter (*p* = 0.643) didn’t show significance. Collagen type I, III, and Aggrecan highlighted no significant difference between the groups (both collagens with *p* = 0.445 and the Aggrecan *p* = 0.4). Conclusion: The scaffold appears to fit as a heterologous implant and did not trigger reactions such as rejection or extrusion of the material into the recipient. However, these results suggested that although the WJ matrix presents several characteristics as a biomaterial for tissue regeneration, it did not display histopathological benefits in trachea tissue regeneration.

## 1. Introduction

The treatment of tracheal pathologies has advanced over the decades but still encounters primary difficulties, such as the extension of the lesion, etiology, location, available technique, material, and the possible formation of stenosis after the procedure [1]. Due to its histological characteristics and modest blood supply to the mucosa, the tracheal tissue is susceptible to ischemic situations, which predisposes it to respiratory mucosal injury [2]. This condition, physiopathologically, can evolve to the development of damage. The two most common types are: (i) tracheal stenosis (narrowing of the trachea) and (ii) tracheomalacia (damage to the cartilaginous tissue of the walls) [3]. 

The treatment depends on the extent and impairment of the site [4]. The most-reported interventions for tracheal reconstruction are due to stenosis of the organ post-intubation or congenital disease, with tracheal stenosis being the most involved condition in this process [5]. Surgical tracheal reconstruction depends on the type and size of the lesion. Gold standard treatment is primary end-to-end anastomosis [6]. 

Viable alternatives for this reconstruction are biocompatible materials that allow cell migration, proliferation, and differentiation, maintaining the integrity of the organ with minimal or no immunological reaction [7]. An ideal material to replace the trachea should have adequate rigidity to avoid collapse, have longitudinal flexibility to preserve the physiological functions, and have lining or enable a lining by vascularized epithelium which would prevent infections and allow adequate healing [8,9]. The WJ presents a greater rigidity in relation to the amniotic membrane and bovine pericardium, besides being more resistant and easier to manipulate because it can be shaped to the structure to be grafted [10].

Within the new set of therapeutic alternatives, the use of decellularized Wharton jelly as a scaffold for the treatment of tracheal defects arises as an alternative that only becomes possible through the development of research in regenerative medicine [11]. The Wharton jelly (WJ) is the main component of the umbilical cord, a structure that connects the placenta to the developing fetus, a mucous tissue formed mainly by hyaluronic acid, collagen, and sulfated proteoglycans [12]. A relevant advantage of its use is that although WJ originates from the embryonic epiblast of the fetal umbilical cord, it is harvested only after birth and not linked by ethical issues, which facilitates its use for experimental studies [11].

This structure can provide a three-dimensional scaffold to support tissue growth due to its biomechanical and biochemical characteristics [13]. Its classification as a biomaterial depends on its biocompatibility, good tolerability by the immune system, no local or systemic toxic effect to the host tissue, and the ability to generate growth factors and cytokines favorable to the regeneration of organs and tissues [14]. In addition, this structure can optimize the formation of new blood vessels for the supply of nutrients, waste transport, and O_2_ in the implanted tissue [15].

In the current study, we analyzed the capacity of tracheal tissue regeneration after the implantation of decellularized WJ in rabbits submitted to a tracheal defect. This was done by comparison of the internal area of the tracheal lumen at the site, the collagen types I and III and the immature cartilaginous tissue located in the center of the lesion. WE achieved the interaction between the tissue, cells and the scaffold but did not activate a response in cell migration or collagen production.

## 2. Materials and Methods

**Figure 1:**
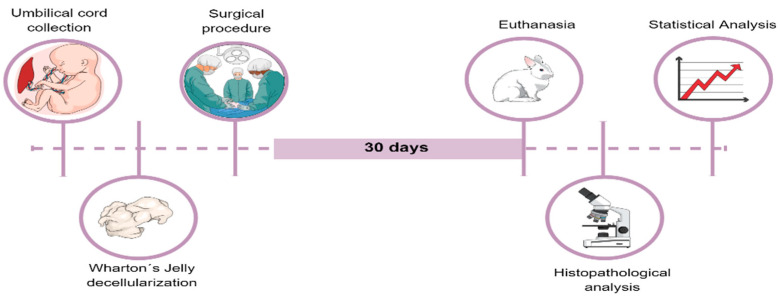
Experimental design: Description of the steps, in which the rabbits were divided into two groups and subjected to a tracheal defect and the implantation of WJ decellularized. Thirty days after surgery, they were euthanized and histopathologically analyzed.

This is an experimental, interventional, and randomized study with 20 New Zealand white rabbits. Animals were randomly divided into two groups: the control group, which only underwent a tracheal defect (*n* = 10), and the WJ group (*n* = 10), which underwent a tracheal defect followed by WJ graft implantation at the tracheal defect site (Figure 1). This project was presented to CEUA (Committee of Ethics in Research in Animal Use of the PUCPR) and approved (Annex 1). All experiments were performed according to the guidelines and the National Institutes of Health Guide for the Care and Use of Laboratory Animals.

### 2.1. Preparation of the Umbilical Cord

The entire process was registered and approved by the Ethics Committee (registration 01616). Two pregnant women (*n* = 2) with gestational age between 36 and 40 weeks who had a vaginal delivery were selected. After the mothers signed the Free and Informed Consent Form, the donated umbilical cords were processed within six to twelve hours after the delivery. The decellularization process occurred at the Pontifical Catholic University of Paraná (PUCPR).

### 2.2. WJ Decellularization

The method using detergents can be ionic or non-ionic. Ionic detergents, such as sodium dodecyl sulfate, are effective in removing nuclear remnants and they can completely solubilize cell and nucleic membranes and fully denature proteins. The amniotic membranes were obtained after cesarean deliveries. Maternal donors were serologically negative for HIV, hepatitis B, hepatitis C, and syphilis. Possible blood clots were immediately removed from the placenta with sterile phosphate buffered saline solution (PBS) (PBS, Invitrogen, Waltham, MA, USA) containing 100 u/mL penicillin and 100 mg/mL streptomycin (Thermo Fisher Scientific, Waltham, MA, USA), until all blood and blood clots were washed away. Amniotic epithelial cells were removed from the human amniotic membrane using SDS (sodium duodecyl sulfate) solution in PBS and incubated with a rotation speed of 100 rpm at 37 °C for 24 h, and finally it was washed more than three times with PBS.

Decellularization was performed aseptically in a BioSAFE class II biological safety cabinet (Veco^®^). For this process, the membranes were removed from the solution (PBS) buffer phosphate pH 7.2 (PBS, Invitrogen, USA) and treated with 0.01% SDS and 0.01% SD (sodium deoxycholate) (Sigma Aldrich, St. Louis, MI, USA) solution for 24 h at 37 °C with the aid of mechanical shaker (Shaking table 109 M, Nova Ética Ltda, São Paulo, Brazil). The membranes were then preserved in PBS at 4 °C according to the methodology described in previous studies [16,17].

### 2.3. Surgical Procedure

All procedures performed on the animals were performed under the s of anesthesia. The animal was positioned in dorsal decubitus. Peripheral venous access was catheterized in the ear with an 20 gauge polyethylene device (Angiocath^®^, Becton^®^ and/or Dickinson^®,^ São Paulo, Brazil). Next, cardiac monitoring with two derivations was installed. Afterwards, the animal was pre-oxygenated with FiO2 of 100%, and anesthetic induction was initiated with propofol at a dose of 5 mg·kg^−1^. 

The surgery started with antisepsis of the cervical region with topical PVPI (povidone-iodine), placement of sterile drapes, and application of local anesthetic with lidocaine 2% without a vasoconstrictor in the lower cervical region (second-third tracheal ring). The approach was through median (longitudinal) cervicotomies, with dissection and preservation of the innervation and vascularization in its lateral portion. Then, a tracheal defect was performed in the anterior region of the trachea, with resection of a 10 mm wide by 20 mm long rectangle. 

Subsequently, the WJ rabbits underwent implantation of a portion of the WJ over the created tracheal lesion. The suture was supported at four repair points, at the ends of the rectangle, with polypropylene thread number “4.0” needled. Once the suture was finished, the pre-tracheal muscles were approximated by continuous suture with the number “zero” needled cotton thread. The graft applied was larger than the tracheal defect performed, and the membrane supported the suture because it was thick with repair points at the edges of the tracheal defect.

**Figure 2:**
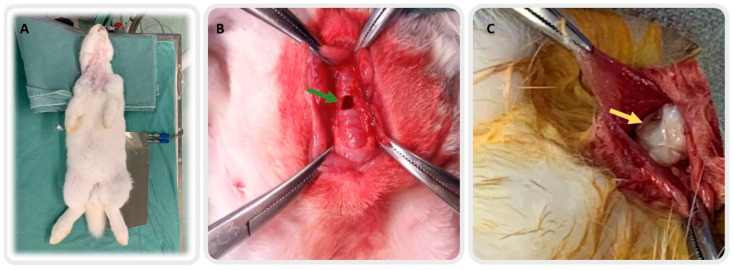
Surgical procedure for tracheostomy. In (**A**) the preparation of the animals for the procedure, in (**B**) the tracheal defect (green arrow) without the implant, and in (**C**) the Wharton’s jelly over the tracheostomy (yellow arrow).

The animals in the control group were only submitted to tracheal defect and the conduit was kept open for healing by the second intention. Approximation of the pre-tracheal muscles and closure of the subcutaneous cellular tissue and skin were also performed. All rabbits underwent the tracheal defect after anesthesia (Figure 2A,B), and the Wharton’s jelly implant group received the scaffold (Figure 2C). The rabbits were analyzed according to some characteristics (weight and the tracheal area), as shown in Table 1. 

Postoperative analgesia for both groups was performed every 8 h until the apparent wound healing using injectable nonsteroidal anti-inflammatory drugs (Carprofen 5–9 mg/mL Pfizer Animal Health^®^) as described by Flecknell (2015). It is well documented that NSAIDs like carprofen inhibit PGE2 synthesis and is the conventional treatment regimen for controlling inflammation pain.

### 2.4. Euthanasia

The animals were sacrificed 30 days after WJ implantation. Survival was 100%. All animals were euthanized using a lethal dose of the anesthetic ketamine (LD50 = 148 mg/kg). For the histological study, the animals were submitted to trichotomy of the region submitted to the procedure and through an incision with a scalpel, the entire skin was removed, along with the injured tracheal tissue.

### 2.5. Histopathology

The samples were placed in labeled plastic containers containing 10% paraformaldehyde and kept aside for 48 h. The materials were sent to the experimental laboratory of pathological anatomy at PUCPR to be processed with the paraffin embedding technique, cut, and stained with Picrosirius Red (SR) staining, and preparation of slides for further histological analysis. After preparing the slides with histological sections, they were scanned using a ZEISS Z1 Axio Scanner slide microscope with ZEN^®^ version 2.6 software, and image analysis was performed using Image-Pro Plus^®^ version 4.5. The image analysis software used a mask in the area of interest. After overlaying the mask and identifying the staining, the software provided the area in square micrometers occupied in each image. After this, it was calculated as a percentage of the ratio of positive staining area per the total area.

### 2.6. Statistical Analysis

Quantitative variables were described by the mean and standard deviation or median and range of variation. The comparison between the two groups was performed using the Student’s t-test in case of a normal distribution or the nonparametric Mann-Whitney test for asymmetric distribution. The significance level adopted was 5% (*p* < 0.05). The data were analyzed using SPSS software version 21.0.

## 3. Results

**Figure 3:**
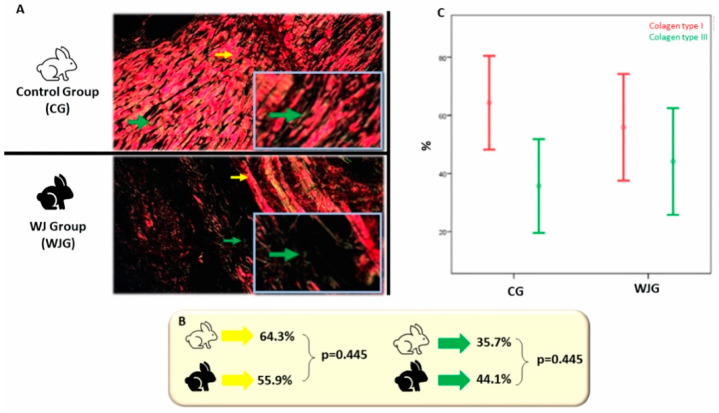
(**A**) Photomicrograph of the control and WJ group with Picrosirius Red (400× magnification). Picrosirius Red stains collagen type I red and collagen type III green, indicated by the yellow and green arrows, respectively. In the highlighted square, it is possible to observe at higher magnification the green arrow marking. (**B**) The mean percentage of collagen type I (yellow) and III (green). (**C**). Mean percentage of collagen types I and III according to the groups. Mature collagen (Type I) is shown in red and immature collagen (Type III) in green in both groups. The sum of collagens is equal to 100 %; *p* values are equal.

The immunohistochemistry technique was applied to identify collagen types I and III with Picrosirius Red (Figure 3A). In the Control group, the mean percentage of type I collagen was 64.3% (±22.6), and in the WJ group, it was 55.9% (±25.7) (*p* = 0.445). The mean percentage of type III collagen was 35.7% (±22.6), and in the WJ group, it was 44.1% (±25.7), also with no statistically significant difference (*p* = 0.445), as can be seen in Figure 3B,C.

**Figure 4:**
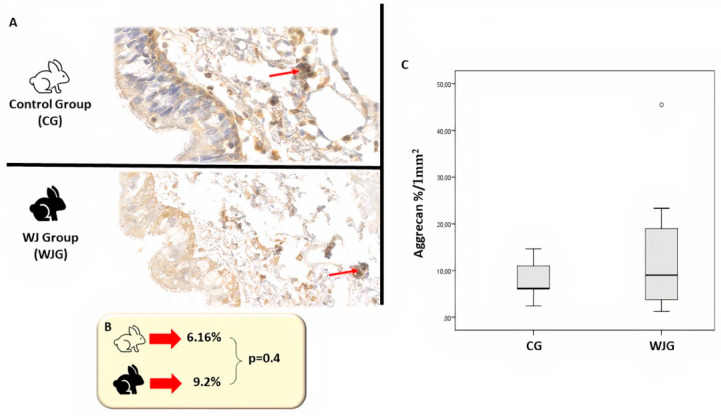
(**A**) Photomicrograph of the control and WJ group with Aggrecan (400× magnification). The red arrows demonstrate the immunoexpression of cartilaginous tissue (brown staining). (**B**) The median percentage of Aggrecan per 1 mm^2^. (**C**) Comparison of the percentage of Aggrecan per 1 mm^2^ according to the group. There was no significant difference between the groups.

Regarding the analysis of the slides concerning cartilage formation, the image of each animal generated a percentage of Aggrecan immunoexpression by calculating the ratio of the area of immunoexpression (1 × 1 mm^2^) by its total tissue area. This value was multiplied by 100 to obtain the percentage of immunoexpression (Figure 4A). In the Control group, the median percentage of Aggrecan per 1 mm^2^ was 6.16% (min: 2.4%; max: 14.6%), and in the WJ group, the median was 9.02% (min: 1.23%; max: 45.5%) (Figure 4B), with no statistically significant difference between the groups (*p* = 0.4), as can be seen in Figure 4C.

## 4. Discussion

The trachea is a complex system that provides tissue support and maintains cell-cell interactions, cell-matrix interactions, cell differentiation, and tissue organization. Despite all efforts, repair of the defect in cartilaginous tissues is unsatisfactory due to the avascular nature and limited regenerative capacity of this cartilage [18]. Several methods have been proposed to replace a defective trachea [1,16,19,20,21,22]. However, an ideal material for tracheal replacement has yet to be discovered. Autogenous cartilage transplantation, allogeneic cartilage transplantation, and artificial substitutes are therapeutic options, but they fail due to source limitations, immunological rejection, exogenous disease transmission, foreign body reactions, and infection [23]. 

In this study, we proposed to use a matrix obtained from decellularized WJ for tracheal reconstruction using a tracheal defect model in rabbits. It suggests the promotion of tracheal repair through the migration of chondrocytes and neighboring epithelial cells. The use of decellularized WJ, like a graft, is based on the characteristics of the extracellular matrix of WJ composed partially of glycosaminoglycans and collagen as the original cartilage [24]. The possibility of this decellularized matrix providing a favorable environment for cell recruitment in the given environment has been analyzed in different ways. Unlike other biomaterials, decellularized WJ proved to be biotolerant and bioinert. It is known that a biomaterial is designated to be bioinert when it integrates with living tissue generating no response, and biotolerable when it does not trigger an immune or inflammatory response at the site of implantation [25]. 

As for the difference in the tracheal area, as shown in Table 1, it was possible to observe that there was no difference when compared after 30 days of follow-up. This material was not able to alter the histological structure of the organ. This aspect is necessary to maintain the function of the trachea. Other studies with different materials, such as a decellularized human amniotic membrane (MAHD) over the tracheostomy in rabbits, showed different responses. The results obtained showed the regeneration of cartilaginous tissue and preservation of the lumen area at the tracheostomy site in the MAHD group [5] Bergonse Neto and colleagues (2018) compared rabbits undergoing tracheostomy with healing by the second intention to a group with porcine small intestine submucosa (SIS) implemented at the tracheostomy site. The results showed no reduction of the tracheal area at the site of the created defect [9]. 

These studies corroborate the results presented since WJ has shown to be a suitable scaffold that provides favorable tissue conditions for the trachea to perform its function, i.e., it does not promote the overproduction of regeneration tissue that alters the structural characteristics of the organ. Unlike synthetic materials, the use of decellularized extracellular matrix scaffolds allows a direct attachment of cells, facilitating the activation of pathways responsible for a cellular response [26]. The presence of growth factors in WJ is the triggering factor for WJ’s differentiation in cartilage. The justification for this fact is that WJ involves the site of tissue injury and allows the migration of native cells near the edge of the trachea lesion toward WJ itself. WJ serves as a framework for tissue damage, and the cells of the trachea grow through WJ stimulated by its growth factors. When analyzing the regeneration of cartilaginous tissue in the region of the implantation of decellularized WJ using the anti-aggrecan antibody, the results did not show a significant difference (graph 2), suggesting that this mediator does not seem to be influenced by the presence of the decellularized WJ, highlighting that the decellularization process should not remove intrinsic growth factors and functional proteins responsible for cell regeneration at the implant site [27]. 

Another possibility is that this biomaterial needs to be modified to have a more satisfactory response within the organism. This modification process is common in metallic implants when, for example, ions are added in the manufacturing process [28]. However, these results can be extrapolated to this structure, in which the addition of growth factors or even undifferentiated cells together with this framework can stimulate migration, the proliferation of cells, and consequently tissue regeneration. Xu, Yong & Duan (2020) presented that decellularized WJ can speed degradation, absorption, and adhesion only when combined with ε-caprolactone and maintained in vivo analysis for a period of 12 months [29]. Another way is to combine the structure with chondrocytes to accelerate tracheal healing [30]. When tissue engineering is associated with tracheal regeneration, three elements must be considered: cells, growth factors, and the scaffold responsible for the tissue regeneration [1]. Achieving the ideal interaction between these three elements has been the challenge in this area of research.

## 5. Conclusions

The study hypothesized that decellularized WJ could be a suitable scaffold for a heterologous implant in tracheal injuries. Although no reactions such as rejection or extrusion of the material in the recipient occurred, it did not induce collagen production and chondrocytes recruitment. It maintained the physical characteristics necessary for the maintenance of tracheal patency, possessing a compatible rigid structure. However, further analysis of this biomaterial is needed to add new insights for possible modifications in its scaffold.

## Figures and Tables

**Table 1:** Characterization of the rabbit population.

	Control Group (*n* = 10)	WJ Group (*n* = 10)	*p*
Weight (kg) *	2.91 ± 0.30	2.77 ± 0.21	0.261
Tracheal area (µm²) *	9,654,271.1 ± 4,687,386.9	10,546,349.7 ± 3,724,181.3	0.643

* Mean and Standard Deviation.

## Data Availability

Not applicable.
